# Barriers and facilitators to uptake of systematic reviews by policy makers and health care managers: a scoping review

**DOI:** 10.1186/s13012-016-0370-1

**Published:** 2016-01-12

**Authors:** Andrea C. Tricco, Roberta Cardoso, Sonia M. Thomas, Sanober Motiwala, Shannon Sullivan, Michael R. Kealey, Brenda Hemmelgarn, Mathieu Ouimet, Michael P. Hillmer, Laure Perrier, Sasha Shepperd, Sharon E. Straus

**Affiliations:** 1 Knowledge Translation Program, Li Ka Shing Knowledge Institute, St. Michael’s Hospital, 209 Victoria Street, East Building, Toronto, ON M5B 1W8 Canada; 2 Epidemiology Division, Dalla Lana School of Public Health, University of Toronto, 155 College Street, Toronto, ON M5T 3M7 Canada; 3 Department of Mechanical and Industrial Engineering, University of Toronto, 5 King’s College Road, Toronto, ON M5S 3G8 Canada; 4 Departments of Medicine and Community Health Sciences, University of Calgary, TRW Building, 3rd Floor, 3280 Hospital Drive NW, Calgary, AB T2N 4Z6 Canada; 5 Département de science politique, Pavillon Charles-De Koninck, Université Laval, Quebec City, Canada; 6 Institute of Health Policy, Management and Evaluation, University of Toronto, Health Sciences Building, 155 College Street, Suite 425, Toronto, ON M5T 3M6 Canada; 7 Research, Evaluation, and Analysis Branch, Ontario Ministry of Health and Long-Term Care, 80 Grosvenor Street, Toronto, ON M7A 1R3 Canada; 8 Nuffield Department of Population Health, University of Oxford, Richard Doll Building, Old Rd Campus, Headington, Oxford, Oxfordshire OX3 7LF UK; 9 Department of Geriatric Medicine, University of Toronto, 27 Kings College Circle, Toronto, ON M5S 1A1 Canada

**Keywords:** Systematic reviews, Determinants of knowledge uptake

## Abstract

**Background:**

We completed a scoping review on the barriers and facilitators to use of systematic reviews by health care managers and policy makers, including consideration of format and content, to develop recommendations for systematic review authors and to inform research efforts to develop and test formats for systematic reviews that may optimise their uptake.

**Methods:**

We used the Arksey and O’Malley approach for our scoping review. Electronic databases (e.g., MEDLINE, EMBASE, PsycInfo) were searched from inception until September 2014. Any study that identified barriers or facilitators (including format and content features) to uptake of systematic reviews by health care managers and policy makers/analysts was eligible for inclusion. Two reviewers independently screened the literature results and abstracted data from the relevant studies. The identified barriers and facilitators were charted using a barriers and facilitators taxonomy for implementing clinical practice guidelines by clinicians.

**Results:**

We identified useful information for authors of systematic reviews to inform their preparation of reviews including providing one-page summaries with key messages, tailored to the relevant audience. Moreover, partnerships between researchers and policy makers/managers to facilitate the conduct and use of systematic reviews should be considered to enhance relevance of reviews and thereby influence uptake.

**Conclusions:**

Systematic review authors can consider our results when publishing their systematic reviews. These strategies should be rigorously evaluated to determine impact on use of reviews in decision-making.

**Electronic supplementary material:**

The online version of this article (doi:10.1186/s13012-016-0370-1) contains supplementary material, which is available to authorized users.

## Background

Knowledge syntheses are comprehensive and reproducible evidence reviews that summarise all relevant studies on a question [1]. They can include traditional systematic reviews and scoping reviews, amongst others. Knowledge translation (KT) focusing on the results of individual studies may be misleading due to bias in their conduct or random variations in findings [2]. Knowledge syntheses that interpret the results of individual studies within the context of global evidence should be considered as the foundational unit of KT, as they interpret the results of individual studies within the context of the totality of evidence and are less susceptible to bias than single studies [3]. Knowledge syntheses, such as systematic reviews, provide the evidence base for implementation vehicles, such as patient decision aids and clinical decision aids clinical practice guidelines and policy [3]. For example, our research team conducts knowledge syntheses for the nationally funded Drug Safety and Effectiveness Network whereby we complete knowledge synthesis to answer questions posed by our provincial and national policy makers [4, 5].

There have been several models or classifications of evidence (or knowledge) use [6–13]. Larsen described conceptual and behavioural knowledge use [7]. Conceptual knowledge use refers to using knowledge to change the way users think about issues. Instrumental knowledge use refers to changes in action as a result of knowledge use. Dunn categorised knowledge use by describing that it could be done by the individual or a collective [8]. Weiss also described several frameworks for knowledge use, including the problem solving model, which she described as the direct application of the results of a study to a decision [9]. She further described this as using knowledge as “ammunition” [9]. Beyer and Trice labelled this type of knowledge use as symbolic, which they added to Larsen’s framework [10]. Symbolic use involves the use of research as a political or persuasive tool. Estabrooks described a similar framework for knowledge use including direct, indirect, and persuasive research utilisation, where these terms are analogous to instrumental, conceptual, and symbolic knowledge use, respectively [11].

We find it useful to consider conceptual, instrumental and persuasive knowledge use [6, 12]. Conceptual use of knowledge implies changes in knowledge, understanding, or attitudes. Research could change thinking and inform decision-making but not change behaviour. Instrumental knowledge use is the concrete application of knowledge and describes changes in behaviour, for example [6]. Evidence can be translated into a usable form, such as a care pathway, guideline, or policy, and is used in making a specific decision. Persuasive knowledge use is also called strategic or symbolic knowledge use and refers to research being used as a political or persuasive tool. It relates to the use of knowledge to attain specific power or profit goals (i.e. knowledge as ammunition) [6, 12].

Use of evidence by policy makers and managers can include any of these approaches. Oliver and colleagues have argued that the concept of knowledge use is further complicated in the policy context because research evidence is just one form of knowledge that informs decision-making. They also pose that researchers need to understand what influences and constitutes policy to better understand what evidence is required and how it can be used [13].

Despite advances in the conduct and reporting of systematic reviews and recognition of their importance in health care decision-making, current evidence suggests that they are infrequently used by health care managers and policy makers [14, 15]. Failure of health systems to optimally use high-quality research evidence results in inefficiencies, reduced quantity and quality of life, and lost productivity [16, 17]. As just one example of this issue, glucose self-testing by older patients with diabetes who use oral hypoglycemic agents has been identified as unnecessary and potentially harmful to patients in systematic reviews [18]. However, financial reimbursement for glucose test strips for these patients continues in many countries, costing just one province in Canada $40 million per year [19].

We previously conducted a knowledge synthesis [20] to identify interventions to encourage use of systematic reviews by health policy makers and health care managers and identified four articles. Three of these articles described one study in which five systematic reviews were mailed to public health officials and followed up with surveys [21–23]. The authors found that 23 to 63 % of survey respondents reported using the systematic reviews to inform policy making decisions. The fourth study was a randomised trial of tailored messages combined with access to a registry of systematic reviews and showed a significant effect on policies made in the area of health body weight promotion by health departments [24]. In more recent systematic reviews [25, 26], no additional studies were identified that assessed interventions to increase uptake of systematic reviews by health care managers and policy makers.

Given that systematic reviews are less susceptible to bias than a single primary study or the opinions of experts, it is not clear why they are not used routinely in decision-making. Two systematic reviews of barriers and facilitators to the use of systematic reviews by any type of decision-maker (e.g. clinicians, patients, managers) identified many factors that contribute to paucity of use including lack of relevance of the questions the reviews are addressing, lack of contextualisation of findings, unwieldy size of the report, and poor presentation format; these factors can be considered intrinsic to the systematic review [25, 27]. The format of systematic reviews has been a key factor identified to influence their use by policy makers and managers [28]. While attention has been paid to enhance the quality of systematic reviews, relatively little attention has been paid to their format. For example, health care managers and policy makers would benefit from highlighting information that is relevant for their decisions including contextual factors affecting local applicability and information about costs [27]. And, because reporting of systematic reviews tends to focus on methodological rigour rather than context, they often do not provide crucial information for decision-makers. Other barriers to use of reviews by health care managers and policy makers include factors extrinsic to the review, such as lack of access and time to seek and acquire systematic reviews and lack of skills to appraise and apply the evidence [27, 29].

Surveys and interviews with policy makers and managers have identified the importance of increasing the usability of systematic reviews in decision-making [30, 31]. Understanding how to make systematic reviews more usable requires consideration of barriers and facilitators to their use, as well as of their format and content. As such, we completed a scoping review on the barriers and facilitators to use of systematic reviews including consideration of format and content by health care managers and policy makers to develop recommendations for systematic review authors and to inform research efforts to develop and test formats for systematic reviews that may optimise uptake. This project arose directly from our decision-maker partners, for whom we conduct knowledge syntheses. This review is part of a multi-phase project to develop and testing a format for a systematic review to optimise use.

## Methods

We conducted a scoping review [32] using guidance from the Joanna Briggs Methods Manual for Scoping Reviews [33]. A protocol was prepared and revised using input from our key stakeholders. Although the PRISMA Statement has not been modified for scoping reviews, we used it to guide reporting [34].

### Data sources and search

We searched the following electronic databases from inception until week 3 of September 2014: MEDLINE, EMBASE, PsycInfo, The Cochrane Database of Systematic Reviews, Cochrane Central Register of Controlled Trials, CINAHL, and LISA (Library and Information Science Abstracts). The literature searches for the previous reviews [20, 26] were peer-reviewed by an information scientist and modified as necessary. The full literature search for MEDLINE is available in Additional file 1, and the other database searches are available from the corresponding author upon request. The search strategy was not limited by study design or language of dissemination. The grey literature was searched using Google after identifying key websites (such as websites of funding agencies and health care provider organisations in Canada, the USA, and the UK that fund or conduct systematic reviews). We supplemented the literature search by scanning references of included articles and relevant, published systematic reviews [20, 25–27, 29]. We also conducted a forward citation search in the Web of Science whereby we used the included studies to identify other potentially relevant studies. The results of the literature search were imported into Synthesi.SR [35], which was used for screening by the review team.

### Study selection: inclusion criteria

Eligible studies included health care managers (defined as an individual in a managerial or supervisory role in a health care organisation with management and supervisory mandates, including public health officials) or policy makers/analysts (defined as an individual (non-elected) at some level of government; they may have some responsibility for analysing data and making recommendations to others and may include regional, provincial, or federal representation) as participants. The focus of the review was on policy/management decision-making; however, clinical decision-making articles were included if policy decision-making was also mentioned and these data could be abstracted. Often the policy articles considered clinical decision-making as well, given that this is often a downstream consideration. For example, if the policy makers felt that clinicians would not implement the evidence, this would be eligible for inclusion. Studies that identified barriers or facilitators (including format and content features) to uptake of systematic reviews by health care managers and policy makers/analysts were eligible for inclusion. All study designs including qualitative or quantitative methodologies where there was a description of the barriers or facilitators to use of evidence from systematic reviews by the relevant end-user groups were eligible. Specifically, we included systematic reviews, experimental (randomised controlled trials, quasi-randomised controlled trials, non-randomised controlled clinical trials), quasi-experimental (controlled before after studies, interrupted time series), observational (cohort, case control, cross-sectional), and qualitative studies. If more than one publication described a single study presenting the same data, we included the most recent. Studies conducted in any setting or country and those published in any language were eligible for inclusion.

### Study selection: screening

To ensure reliability, a calibration exercise with reviewers was conducted prior to commencing screening. Using the eligibility criteria, a random sample of 10 % of citations from the search were screened independently by all reviewers. Screening only began when percent agreement was >90 % across the review team. A similar calibration exercise was completed prior to screening full-text articles for inclusion. Subsequently, two reviewers independently reviewed titles and abstracts and full-text articles for inclusion. Conflicts were resolved through discussion.

### Data abstraction

Two reviewers independently reviewed each full-text article and extracted relevant data. Data were extracted on study design, participants, country, barriers, and facilitators to use of the systematic review. Differences in abstraction were resolved by discussion. We did not assess risk of bias of individual studies because our aim was to map the evidence, as is consistent with the proposed scoping review methodology [32, 33].

### Data charting and collation

The barriers and facilitators were charted using a taxonomy of barriers and facilitators to implementation of clinical practice guidelines by clinicians [36]. This taxonomy was expanded to include attributes of the systematic review, specifically its format and content, and was reviewed by a health care manager to ensure face validity. The taxonomy was based on a systematic review of barriers and facilitators to evidence use by clinicians as no similar review was available explicitly for policy makers and managers at the time we completed our review. We shared the taxonomy with our decision-maker partners to assess for face validity and no additional categories were identified. Two reviewers reviewed each article and identified the unit of text relevant to each of these factors using a coding scheme they developed. Qualitative analysis was conducted using NVivo 10 [37]. Codes were aggregated by themes, centred on whether the barriers/facilitators influenced participants’ attitudes towards; knowledge of; skills in seeking, appraising or using; or use of systematic reviews in decision-making. Discrepancies in coding were discussed by the team to achieve consensus.

### Consultation

Team members (including representatives from the health care managers and policy makers/analysts from the Ontario Ministry of Health and Long-term Care) were consulted at various stages of the scoping review to provide input on the search, data abstraction, and interpretation of the results.

## Results

### Literature search

**Fig. 1:**
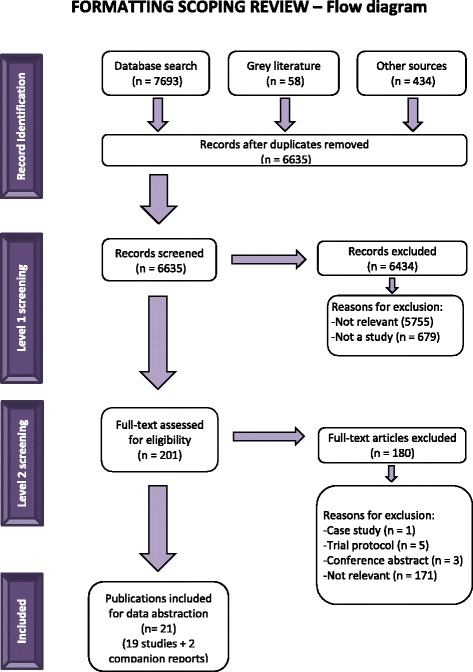
Study flow diagram

A total of 6635 titles and abstracts and 201 full-text articles was assessed for eligibility. Subsequently, 19 studies reported in 21 publications fulfilled the eligibility criteria and were included [21–24, 30, 31, 38–52]. The reasons for excluding full-text articles are provided in Fig. 1.

### Characteristics of the included articles

All but one of the studies was published after 2000. Authors of the papers were commonly from Canada, Australia, and the UK. Nineteen of the publications were qualitative studies, 1 study was quantitative and described the impact of an intervention to facilitate use of systematic reviews, and 1 was a systematic review. A description of included studies is provided in Table 1.

**Table 1:** Study Characteristics of Included Studies

Studies [reference]	Country	Study design	Setting	Type of end user or participant	Number of end users or participants
Albert 2007 [38]	Mali	Qualitative study	National government: pharmaceutical decision-making group	Health policy maker	19
Armstrong 2012 [39]	Australia	Qualitative study	Health related organisations	National stakeholders of relevance to employment and health, advocacy, member organisation for a workforce or national policy	9
Atack 2010 [40]	Canada	Qualitative study	Different health care settings (research hospital, community hospital, community health centre, long-term care facility, district/regional health authority, government ministry/department, academic health science centre, others)	Health care manager	42 for Technology Acceptance Model survey (TAMS) and 12 for interviews
Campbell 2009 [42]	Australia	Qualitative study	Public health and health service government	Health policy maker and researcher	38 policy makers/41 researchers
Campbell 2011[41]	Australia	Qualitative study	Independent research organisations and Department of Health	Health policy maker and researcher	8 policy makers/11 researchers
Ciliska 1999 [21]	Canada	Qualitative study	Public health organisations	Health care managers and total, 277 were eligible, 242 participated in the first survey; 225 participated in the second survey	
Dobbins 2001a (CR to Ciliska 1999) [22]	Canada	Qualitative study			
Dobbins 2001b (CR of Ciliska 1999) [23]	Canada	Qualitative study			
Dobbins 2004 [30]	Canada	Qualitative study	Public health/health promotion, government	Health care managers and policy makers	46
Dobbins 2004 [31]	Canada	Qualitative study	Public health (programme managers, directors, epidemiologists, medical officers of health, provincial consultants, local board of health members)	Health care managers and policymakers	51 participants
Dobbins 2007 [43]	Canada	Qualitative study	Public health decision-makers: managers, directors, medical officers	Health care managers and policy makers	16
Dobbins 2009a [24]	Canada	RCT	Public health departments	Health care manager	108
Jewell 2008 [44]	USA	Qualitative study	Conference attendees	Health policy maker	28 (11—public health; 15—legislators; 2—both)
Lavis 2005 [45]	Canada, Scotland, Norway, UK	Systematic review and qualitative study	Studies of decision-making by health care managers and policy makers, interviews of managers, policy makers, website review	Health care managers and policy makers	17 studies and 29 participants
Packer 2000 [52]	England	Qualitative study	5 health authorities	Health policy makers: health authority contacts including public health consultants, public health specialist registrars, a research analyst, and a public health nurse	11 health authority contacts
Ritter 2009 [46]	Australia	Qualitative study	Drug policy units	Health policy maker	31 participants
Rosenbaum 2011 [47]	Norway, Argentina, China, Colombia, South Africa, and Uganda	Qualitative study	National or international health service or policy-related work in health departments, national insurance programmes, hospitals, or aid organisations	Health care managers and policy makers	18 participants
Shepperd 2013 [48]	England	Qualitative study	Regional health authorities	Clinicians and Commissioners (including 2 general practice commissioners, 3 pharmacists, 6 public health consultants, and 4 health caremanagers)	23 clinicians; 15 commissioners (2 general practice commissioners, 3 pharma, 6 public health consults, 4 managers)
Suter 2011 [49]	Canada	Qualitative study	Provincial health authority	Health care managers and policy makers	13 participants
Vogel 2013 [50]	Canada, England and Wales, Finland, Germany, Italy, the Netherlands, Norway, Scotland, and Spain, Cameroon, Ethiopia, Mozambique, Zambia, Uganda, and South Africa	Qualitative study	National policy organisations	Health care managers and policy makers	112 respondents
Yousefi-Nooraie 2009 [51]	Iran	Qualitative study	Capacity building workshop on systematic review methods in Iran	Health care managers and policy makers	131 participants

*CR* companion report, *RCT* randomised controlled trial

### Barriers to use of systematic reviews

Barriers to the use of systematic reviews are presented in Table 2. We have also presented the results per study identified and present this in Additional file 2.

**Table 2:** Barriers to use of systematic reviews by policy makers and health care managers

Attitudes			Knowledge	Skills	Behaviour	
Lack of agreement	Lack of outcome expectancy	Inertia of previous or actual practice/lack of motivation	Lack of awareness/familiarity	Lack of skills	SRs/intrinsic factors	Extrinsic factors
Limited quantity of research on topics of importance to them, e.g. economic impact, emerging technologies [21, 44, 50]	Avoid providing specific recommendations for action based on SR; do not believe in causal linkage [45]	Lack of motivation to use reviews will require changing policy makers’ attitudes [51]	Administrators’ limited understanding of the function of SRs [44]	Participants’ poor conceptual understanding of systematic reviews [47]	The review itself did not appear to be user-friendly due to inaccessible language and dense layout [39]	Policy inconsistency within health care system (differing interests, different policies run in parallel, performance based incentives) [48]
Systematic reviews do not necessarily frame the existing evidence in terms of their policy applications [44]			Information overload leads to lack of awareness of a particular systematic review [24, 43]	The health agency staff had not been taught “to continue to use research to inform their decisions, to inform their practice. They therefore made decisions based on “common sense”, “gut level”, “standards of practice”, and comparative convenience and awareness of available data, rather than based on systematic reviews of research.” [44, 52]	Accessibility: within a systematic review, have difficulty identifying key messages [44]	Attacks on an evidence-based approach. Several officials also discussed instances in which the whole notion of evidence-based health care had come under direct attack, usually by pharmaceutical companies, sometimes in collaboration with advocacy groups, some of which hid their involvement with industry [44].
Lack of or unknown credibility of authors of the research [21]			No policy makers mentioned having utilised information from systematic reviews, and most seemed unaware of their existence [38]	Lack features that would make them easier for government officials to evaluate. For example, the quality of studies is often difficult for non-experts to interpret because the explanation of research methods is long and complicated [44]	Concepts presented in tables, including those that showed the GRADE assessment and different levels of risk, were not clear [47]	Accessibility. Even when evidence is available, policymakers may have problems obtaining it [44]
Ethical disagreement [21]				Appraising and synthesising the evidence was seen as an even bigger challenge [40]	Tables running over 2 pages were cumbersome to read [47]	Lack of availability of research results [21]
Research information not valued at community level [21]				Difficult to understand by people not trained in evidence-based medicine [21, 47]	Abbreviations caused confusion [47]	Lack of resources to implement research [21]
Policy decisions are made based on other factors like cost and equity considerations, particularly if evidence base is frail [52]				Words like “sample size” and “relative risk” would be difficult to interpret [47]	Use of jargon and/or unfamiliar vocabulary [47]	Policy climate—provincial/regional not conducive to use [21]
Mismatch between the type of content offered and their information needs [47]				Lack of expertise in evaluating SRs [44]	Numbers in the text and those in the tables do not correspond precisely [47]	Lack of timely completion of review [21]
Translating evidence to the local context (including sub groups of patients): individuals frequently had to make independent decisions about how to relate evidence to the needs of their local context, discuss and debate the evidence with local stakeholders and take decisions about its usein practice [48]				Appraising and synthesising the evidence was seen as an even bigger challenge [40]		Current practice patterns lead policy makers and managers reluctant to use reviews [21]
Policymakers expected content lying outside the scope of a review: recommendations, outcome measurements not usually included in a review, detailed information about local applicability or costs, and a broader framing of the research enquiry [47]					Wanted a shorter, clearer presentation [47]	Cost of retrieving information prohibitive [21]
					Reviews covered issues at a more complex level than required [52]	Insufficient authority to implement research results [21]
					Lack of detail on how to use strategies, tools, processes that would lead to successful integration (i.e. guidance on breaking down systems barriers or how to achieve integration in the context of big, complex system) [49]	Policy makers had difficulty finding brief research summaries and systematic reviews when they were needed (i.e. difficulty accessing SRs) [42]
					Tended not to use the full report instead referring to the less dense, more accessible articles [49]	Limited time to find, retrieve, read, and translate research [21, 24, 47]
					Wanted a shorter, clearer presentation [47]	Research often published in academic sources, poorly accessible to policy makers, LMIC policy makers have limited access to subscription-based K or the internet [47]
						Lack of indexing local journals in international databases, harmonised reporting criteria, editorial processes and presentation of local journals, minimum standards for reporting of research for all local journals. Coverage and searching quality of databases of papers published in local language needs improvement, single national database for research registration, technical and professional support for current databases [51]
						If department within commissioning organisation is not in a position of strength, unlikely that evidence will be used for decision-making [52]
						Lack of time to find or discuss evidence, usually need an answer to a problem on the same day [52]
						Finding the evidence was described as problematic. Several fellows called for greater access to systematic reviews; this was a resource they wanted to see augmented through the desktop [40]
						Policy makers’ belief that searching, accessing, and reviewing research findings are highly time consuming is perhaps a good argument for the increased production, promotion, and dissemination of systematic reviews [38]
						Limited time to read full study reports (of a SR) [43]

*SR* systematic review

#### Attitudes

Factors limiting the use of systematic reviews through an affective component were considered as those barriers that affected attitudes towards the use of systematic reviews [36]. Lack of agreement with the usefulness of systematic reviews in general and lack of agreement with results of specific systematic reviews were identified as such barriers [21, 44, 50]. With regard to the former, participants believed that systematic reviews may challenge their autonomy in decision-making because the evidence in the review would dictate their decisions and this was perceived to be a barrier to their use. Lack of outcome expectancy was also a barrier to use whereby participants believed that decisions based on systematic reviews would not lead to the desired outcome because they did not believe the causal inference implied by the results of the review [45]. Lack of motivation to change or resistance to change was a barrier to use of systematic reviews [51]. Barriers related to lack of agreement with results of specific systematic reviews were also noted and included features such as participants’ lack of agreement with the evidence interpretation, their lack of confidence in the authors of the review, or their belief that the results of the review are not valid [21].

#### Knowledge

Factors limiting adherence through a cognitive component were considered knowledge barriers to use of systematic reviews [36]. For example, participants reported the lack of awareness or lack of familiarity with a systematic review as influencing use. Particular challenges related to this factor included the tremendous volume of information required for participants to stay abreast of in relevant areas, the lack of knowledge on how to access relevant systematic reviews, and the lack of awareness of the importance of systematic reviews [24, 43, 44].

#### Skills

Factors limiting adherence to systematic review evidence through a lack of ability were considered barriers related to skills [36]. Participants reported the lack of skills to find, assess, interpret, or use systematic reviews in decision-making [44, 47, 52]. Additional elements included the lack of ability to reconcile patient preferences with recommendations.

#### Behaviour

Several behavioural barriers to use of systematic reviews were reported, and these focused on external barriers to their use, including patient and clinician factors. For example, patient and clinician resistance to implementing the evidence outlined in the systematic review may lead to policy makers and managers being reluctant to use the evidence. Participants also reported factors intrinsic to the systematic review as barriers to their use, including the presence of contradictory results from different systematic reviews or difficulty accessing reviews and in particular, difficulty identifying their key messages quickly when they are needed for decision-making [39, 44, 47]. Extrinsic or environmental barriers were identified including lack of time and organisational constraints, which prevent the individual from implementing the review. In one of the intervention studies that were included, lack of time and availability of relevant systematic reviews were identified as barriers to use in decision-making by public health officials [21, 42, 44].

### Facilitators to use of systematic reviews

Facilitators to the use of systematic reviews are presented in Table 3.

**Table 3:** Facilitators to use of systematic reviews by policy makers and health care managers

Attitudes		Knowledge	Skills	Behaviour	
Agreement/usefulness	Motivation	Awareness/familiarity	Expertise/experience/training	SRs/intrinsic factors	Extrinsic factors
Stakeholders described potential uses of the review as being more indirect (creating a culture); for example, for advocacy purposes internally, to promote a particular intervention approach and to identify gaps of where further evaluation was needed [39, 41, 42]	Expecting to use the systematic reviews in the future [23]	Recognition of relative importance of SR compared to other sources of information such as single studies (culture of evidence-based decision-making) [31]	One’s age [23]—younger, more likely to use	Delineating the effects for a particular group with more focused subgroup analyses in SRs [44] enhanced their usefulness	Making decisions in collaboration with other community organisations increased likelihood of using reviews [23]
SRs to provide guidance and suggestions for implementation of findings, not just reporting facts [43]	Willingness of health care providers to use systematic reviews [51]		Number of years since graduation [23]—more recent graduates more likely to use	Providing information about the benefits, harms (or risks), and costs [45]	Increasing the opportunities for interaction and exchange between policy makers and researchers is key to promoting the use of research evidence in policy [42]
Most policy makers reported having needed the data and reviews in the past 12 months, having commissioned research or reviews during this period, and having used evidence to contribute to the content of policy [42]	Perception that reviews facilitate critical appraisal of evidence and are easy to use, information about what works and clearly articulated implications for policy (costs, applicability, impacts on equity) [47]		Providing training in basic search skills may increase use [52]	Concise statements about lives or money can infuse the political discussion with a tone of rationality, framing the trade-offs as technical and straightforward [44]. Providing information about the benefits, harms (or risks), and costs [45]	One-to-one interaction with the researcher to discuss research findings [43]
Respondents who expected to use the reviews in the future were more likely to have used a review than those who did not expect to use the reviews [22]	Presenting selected important systematic reviews to policy makers may change their attitudes towards evidence-based decision-making, presenting successful/unsuccessful policies [51]		Perception that systematic reviews could overcome the barrier of limited critical appraisal skills [22]	Identify attributes of the context in which the research included in a systematicreview was conducted to inform assessments of the applicability of the review in other contexts [45]. Concise statements about lives or money can infuse the political discussion with a tone of rationality, framing the trade-offs as technical and straightforward [44]. Providing information about the benefits, harms (or risks), and costs [45]	Organisational research culture [23, 24] favouring use of research to inform decision-making
Coming from credible sources [47]			Ongoing training in critical appraisal of research literature [23]	Add additional local value toany type of systematic review by using language that is locally applicable and by engaging in discussions about the implications of reviews with the health care managers and policy makers who could potentially act on the reviews’ take-home messages [45]. Identify attributes of thecontext in which the research included in a systematic review was conducted to inform assessments of the applicability of the review in other contexts [45]. Concise statements about lives or money can infuse the political discussion with a tone of rationality, framing the trade-offs as technical and straightforward [44]	Fund production and updating of SRs with additional resources for health care managers and policy makers to interact and fund local adaptation process for SR [45]
Relevance to policy decisions [31] Most policy makers reported having needed data and reviews in the past 12 months, having commissioned research or reviews during this period, and having used evidence to contribute to the content of policy [42]			Opportunities for training and education on systematic reviews (definition, significance, appraisal) [30] Ongoing training in critical appraisal of research literature [23]	Ensure SRs are included in a one-stop-shop that provides quality-appraised reviews [45]. Add additional local value to any type of systematic review by using language that is locally applicable and by engaging in discussions about the implications of reviews with the health care managers and policy makers who could potentially act on the reviews’ take-home messages [45]. Identify attributes of the context in which the research included in a systematic review was conducted to inform assessments of the applicability of the review in other contexts [45]	Collaborative creation of knowledge in a format that is easy to view and comprehensible and allows fast and easy referencing [49]
Reassurance that no reviews have been missed [52]. Respondents who expected to use the reviews in the future were more likely to have used a review than those who did not expect to use the reviews [22]			Opportunities for training and education on systematic reviews (definition, significance, appraisal) [30]	Replacing unfamiliar terms or adding definitions to the re view [47]. Ensure SRs are included in a one-stop-shop that provides quality-appraised reviews [45]. Add additional local value to any type of systematic review by using language that is locally applicable and by engaging in discussions about the implications of reviews with the health care managers and policy-makers who could potentially act on the reviews’ take-home messages [45]	Involvement of librarians and health information specialists as a solution to lack of database access, establishment of a national portal for expanding access [51]
Coming from credible sources [47]			Teaching about systematic reviews, integration of this course into postgraduate educational curricula, mandatory education of research methods to researchers, consultation support in methodology and scientific writing, professional methodologists on research teams [51]	Provide section on the relevance of the evidence and the intervention for low and middle income countries (LMICs) [47]. Ensure SRs are included in a one-stop-shop that provides quality-appraised reviews [45]	Involvement in an advisory role by policy makers on research teams (i.e. involved with the development of research questions, assisted with dissemination) [42]
Relevance to policy decisions [31]			Perception that systematic reviews would overcome the barrier of not having enough time to use research evidence [23]	Make the user-friendly “front end” of systematic reviews available through an online database that could be searched using keywords that make sense to health care managers and policy-makers and that is linked to the full reviews when they are available through other sources, such as The Cochrane Library [45]. Replacing unfamiliar terms or adding definitions to the review [47]	Position of end user within organisation/system: programme manager vs. director vs. medical officer differed in uptake of SRs [22]
Reassurance that no reviews have been missed [52]				Use of stories to help integration come alive for participants so they could see how it lives operationally [49]. Provide section on the relevance of the evidence and the intervention for low and middle income countries (LMICs) [47]. Replacing unfamiliar terms or adding definitions to the re view [47]	Value the organisation placed on using research evidence for decision-making [23]
				Make the user-friendly “front end” of systematic reviews available through an online database that could be searched using keywords that make sense to health care managers and policy makers and that is linked to the full reviews when they are available through other sources, such as The Cochrane Library [45]. Provide section on the relevance of the evidence and the intervention for low and middle income countries (LMICs) [47]	Having direct access to online database searching [23]
				Use of stories to help integration come alive for participants so they could see how it lives operationally [49]. Make the user-friendly “front end” of systematic reviews available through an online database that could be searched using keywords that make sense to health care managers and policy makers and that is linked to the full reviews when they are available through other sources, such as The Cochrane Library [45]	Existence of mechanisms to facilitate transfer of new information in health unit [23]
				Use of less dense and more accessible articles [49]. Use of stories to help integration come alive for participants so they could see how it lives operationally [49]	Reallocate funding away from single study knowledge transfer strategies, fund rapid reviews, more proactive knowledge transfer, health care manager [45]
				Removing jargon and using language that is locally applicable, engage in discussion about the potential implications of the review [45]. It must be packaged to incite and persuade, “to translate the evidence into something that is understandable by the average legislator, average citizen” [44]. Concrete recommendations for practice [31]	Priority of and support for systematic reviews [51]
				It must be packaged to incite and persuade, “to translate the evidence into something that is understandable by the average legislator, average citizen” [44]	Announce priorities to be addressed using SR [51]
				Use of familiar, non-jargon language recommended [47]. Removing jargon and using language that is locally applicable, engage in discussion about the potential implications of the review [45]	Meeting requestors time constraints [52]
				Reassurance that no reviews have been missed [52]. Use of familiar, non-jargon language recommended [47]. Removing jargon and using language that is locally applicable, engage in discussion about the potential implications of the review [45]	Consistency in follow-up of individuals using on-demand service to appraise and interpret reviews of research [52]
				Easy to use [31]. Framing the evidence in terms of how they can implement it (specifically as a list of questions to be considered when developing and implementing an integrated health system, information about how to engage stakeholders, build relationships and communicate appropriately across target audiences) [49]. Reassurance that no reviews have been missed [52]	Researchers and policy makers generally found reviews commissioned through evidence check to accurately reflect the state of the evidence, implying that the requirement for rigour and comprehensiveness was not unnecessarily compromised by the rapid timeframe in which the reviews were conducted. It is likely that this is due to both knowledge brokers’ attempts to assist in precisely defining the focus and scope of reviews early in the commissioning process, and researchers’ depth of content knowledge and methodological expertise [41]
				Easy to use [31]. Framing the evidence in terms of how they can implement it (specifically as a list of questions to be considered when developing and implementing an integrated health system, information about how to engage stakeholders, build relationships and communicate appropriately across target audiences) [49]	
				Using consistent language and standard phrases to describe effect sizes and the quality of the evidence [47]. Easy to use [31]	
				Using consistent language and standard phrases to describe effect sizes and the quality of the evidence [47]	

#### Attitudes

Participants identified that agreement with the usefulness of systematic reviews, belief in their relevance, and their applicability to policy facilitated their use. Participants perceived systematic reviews were useful if they had confidence in the review authors [47, 52]. Enthusiasm and motivation to change were facilitators for use of systematic reviews; in particular, if important and relevant reviews could be provided to policy makers at key points in decision-making, this was perceived to be influential in their further use [23, 47, 51].

#### Knowledge

Familiarity or awareness of systematic reviews were potential facilitators of their use. In particular, knowledge of their importance relative to single primary studies [31].

#### Skills

Participants reported that skills in seeking, appraising, and interpreting systematic reviews facilitated their use [22, 23, 30]. For example, training in basic searching skills was identified as a facilitator [52].

#### Behaviour

Extrinsic factors that were perceived to facilitate use included creating collaborations between policy makers and researchers whereby researchers could provide systematic reviews of relevance to policy makers in a timely fashion and facilitate their interpretation [23, 24, 42, 43]. This approach reflects a change in culture for researchers and policy makers/managers. In one of the intervention studies that were included, resources to implement the research and availability of the systematic review were identified as facilitators to using them in decision-making by public health officials [21].

### Format features to facilitate use of systematic reviews

Several recommendations from policy makers and health care managers regarding formatting of systematic reviews to enhance uptake were identified (Table 4). Many participants suggested a one-page summary of the review including clear “take home” messages written in plain language, the publication date of the review, and sponsoring logos [47, 49]. Some participants recommended that the summary include sections on relevance, impact, and applicability for decision-makers [45, 47, 52]. They also recommended that the report for the full review should use a liberal amount of white space with bullet points (avoiding dense text) and simple tables (less than one page in length) and consider tailored versions with targeted key messages for relevant audiences [24]. Another suggestion was to frame the title of the systematic review as a question [47].

**Table 4:** Formatting features of systematic reviews to enhance uptake

Summary	Dissemination of SRs	Layout, presentation, setup
Summary statement [30]	Share material on a website [24, 43]	Graded format with key messages [47]
1-page summaries in plain language [49]	Provide tailored, targeted messages for relevant audiences [24]	Recipe type guidance, the information indicates this, this, and this [52]
Abbreviated format of research evidence, such as an executive summary, would be preferable (1 to 2 pages long ) [43]	Electronic communication channels are generally preferred [43]	Title framed as a question [47]
Expectations of short, clear summary [47]	Newsletters containing summaries of current research developed and directly emailed to managers [43]	Reformatting the text to make it easier to pick out important parts [47]
Boxes placed throughout the summaries [47]		Chart on first page describing what review is about [47]
Summary of findings tables [47]	Reports could be either distributed through professional organisations or through a clearinghouse [43]	A modified academic abstract (relevance and description of review characteristics including the impact, applicability to setting, costs, or other considerations and need for no further evaluation) [47]
1-page summaries with references, so the reader is able to investigate further, and case studies [49]	Active delivery of information (as opposed to access to online registry) [24]	Preference for less dense, more accessible literature [49]
		Wanted a shorter, clearer presentation [47]
		A bullet point evaluation or rating system of study design quality so that for those of us who do not make our living doing that, we do not have to read a half dozen pages to ferret it out [44]
		Develop a more user-friendly “front end” for potentially relevant systematic reviews (e.g. 1 page of take-home messages and a 3-page executive summary) to facilitate rapid assessments of the relevance of a review by health care managers and policy makers and, when the review is deemed highly relevant, more graded entry into the full details of the review [45]
		Well written and concise [47]
		Limiting the number of tables and not letting them break across pages [47]
		Simplifying the text and tables and ensuring that the results in the text matched those in the tables [47]
		Moving partner logos and the summary publication date to the front page [47]

### Content features to facilitate use of systematic reviews

A commonly requested feature amongst the studies was to frame the evidence in terms of policy application, including implications of implementation and potential outcomes (Table 5) [43, 47, 49]. Participants suggested that the methods details be minimised to focus on the critical elements and that the bulk of the report should focus on the results and interpretation [43, 44, 47]. Ways to make study quality of included studies easy for users to interpret, such as providing a graphical summary, were suggested [44, 47, 52]. Participants also asked that consistent approaches be used to report effect sizes of interventions throughout the review report.

**Table 5:** Content features of systematic review that may increase their use

Decision-making focus	Easy to understand	Details on included studies
Address relevant policy questions not academic or business focused questions [44]	Information about the information or meta-information that tells you what to expect [47]	Provide rating scale for quality of study design [44]
Clearly articulate the implications of the findings to public health practice and policy [43]	Include content that was focused on key findings or the “bottom line” from the study [43]	Include section on the relevance of the evidence and the intervention for low and middle income countries (LMICs) [47]
Provide potential short- and long-term outcomes expected as a result of implementing the research findings into practice [43]	Provide references to more detailed findings so the reader is able to investigate further if needed [49]	Include table describing the characteristics of the reviews [47]
Policy makers expect content lying outside the scope of a review: recommendations, outcome measurements not usually included in a review, detailed information about local applicability or costs, and a broader framing of the research enquiry [47]	Lack features that would make them easier for government officials to evaluate. For example, the quality of studies is often difficult for non-experts to interpret because the explanation of research methods is long and complicated [44]	Include critical appraisal of included studies [52]
Frame the evidence in terms of how they can implement it (specifically as a list of questions to be considered when developing and implementing an integrated health system (which was topic of the review in this study), information about how to engage stakeholders, build relationships, and communicate appropriately across target audiences) [49]	Replace the section for references with a section for “additional information”: information that was helpful for understanding the problem, that provided details about the interventions, or that put the results of the review in a broader context [47]	Include bullet point evaluation or rating system of study design quality so that “for those of us who don’t make our living doing that, we do not have to read a half dozen pages to ferret it out” [44]
	It must be packaged to incite and persuade, “to translate the evidence into something that is understandable by the average legislator, average citizen” [44]	Provide table describing the characteristics of the reviews: makes clear what the review was looking for [47]
	References are clear [47]	
	Use familiar, non-jargon language [47]	
	Use consistent language and standard phrases to describe effect sizes and the quality of the evidence [47]	
	Limit the discussion of methods [43]	

## Conclusions

We identified several determinants of the use of systematic reviews by policy makers and managers including factors influencing attitudes, knowledge, skills, and behaviours. For authors of systematic reviews, there are factors that are potentially modifiable and that may increase use of systematic reviews including features affecting format and content. From a format perspective, review authors can consider providing a one-page summary with key messages including importance of the topic, key results, and implications for decision-makers. This summary should be clearly written and concise. Similarly, the report for the full review should use white space, avoid dense text, and try to limit tables to one page. With regard to content of the reviews, the methods should be concise and the results should provide an easy to interpret summary of the risk of bias of individual studies, keeping in mind that the audience may have limited skills in appraising the evidence and limited time to do so. The use of a graphical display of the risk of bias, such as the figure advocated by the Cochrane Collaboration [53] is something review authors should consider using. The discussion should include the relevance of the results to decision-makers and factors important for contextualising the evidence. Systematic reviews should include consideration of what factors influence contextualisation of the evidence. To make knowledge more useful to the local context, commissioners of reviews such as policy makers frequently undertake processes to contextualise evidence [54], and if guidance on this can be provided by researchers, this may facilitate the process. For example, if information is available on how the evidence might be useful in resource constrained circumstances versus higher income settings, this should be provided in a systematic review. To make knowledge more useful to the local context, commissioners of reviews, such as policy makers, frequently undertake processes to contextualise evidence [54], and if guidance on this can be provided by researchers, this may facilitate their efforts. It was also suggested that the messages be tailored to different audiences, reflecting their needs. For example, the summary and report that is sent to policy makers would be different from the one sent to health care managers. These formatting suggestions should also be considered by journal editors and publishers to consider enhancing use of reviews. Of particular importance is the topic addressed by the systematic review as several studies raised the concern that the topics often were not perceived to be relevant by policy makers and managers. As such, it was suggested that a different approach be undertaken to conduct reviews whereby partnerships between researchers and decision-makers are created to ensure that the questions the reviews are tackling are relevant to the decision-makers. This approach requires a change in the organisational culture within health care and research, although there are numerous examples of successful partnerships like these [4, 5, 55, 56].

Several factors were perceived to influence use of systematic reviews that are extrinsic to the review, including a lack of motivation to use them, lack of awareness, and lack of skills to seek, appraise, and interpret systematic reviews. Tackling these challenges could also be addressed by developing partnerships between researchers and decision-makers, such as a train the trainer approach whereby systematic reviewers work with decision-makers to build capacity in either conducting reviews or interpreting their results within their organisation. If researchers can provide useful systematic reviews and illustrate how they can be used in a timely fashion to inform decision-making, this could provide motivation for continued use. Similarly, strategies to enhance awareness of reviews could be enhanced by these partnerships. Participants also raised the concern that using a systematic review to guide decision-making led to a perceived lack of autonomy in decision-making. This concern has been raised by clinicians for many years in relation to the practice of evidence-based health care, reflecting the issue that using evidence implies a “cookbook” approach to decision-making [57]. It highlights a misunderstanding around the appropriate use of evidence, whereby the practice of evidence-based health care requires integration of evidence, expertise, and values and circumstances [58].

Our results are consistent with systematic reviews of barriers [29] and facilitators [27] to use of systematic reviews by any decision-maker. These same authors also recently published a systematic review of interventions to increase use of systematic reviews [25]. Oliver and colleagues also published a review of barriers and facilitators to use of evidence by policy makers; however, their review was not limited to use of systematic reviews [59]. Search dates for all of these systematic reviews were between 2010 and 2012, while ours was extended to 2014. We found an additional six to nine studies not included in these reviews. Moreover, our review focused on factors (and categorised them) including format and content of the review. Our intent is to use the results of this review to inform the development of a template for providing results of systematic reviews to decision-makers and as such, we wanted to extend the findings of other reviews to include both intrinsic and extrinsic factors. Because of this focus, we did not include use of other types of research evidence beyond systematic reviews (e.g. results of single studies). Of note, we identified no additional studies reporting interventions to increase use of systematic reviews.

There are several limitations to our scoping review. First, it is a scoping review because we wanted to map the literature to inform future research on formatting systematic reviews and to provide guidance for authors of systematic reviews. As such, we did not perform risk of bias assessment on individual studies [32]. Second, most of the studies that were included in our scoping review were small qualitative studies and thus their results may not be generalizable. However, studies from a broad range of countries were included in our review, and the results are consistent across studies and previous reviews. Third, the literature search on this topic is limited by poor indexing of the primary studies in this area. To overcome this, our comprehensive search of the databases was supplemented by a grey literature search.

This review represents the first phase of a multi-phase project with is being conducted in partnership with decision-makers from four provinces in Canada. The next phases include completing a survey of perceptions of barriers and facilitators to use of systematic reviews by policy makers and health care managers in these provinces; integrating the survey and review results to develop a format for systematic reviews and test its usability using heuristic and individual usability testing; and conducting a randomised trial to assess a traditional systematic review format compared with the new format on the ability of health care managers and policy makers to understand the evidence in the review and apply it to a relevant health care decision-making scenario. We have done similar work to create a format for clinicians and found that it influences their ability to apply the evidence from a systematic review to a clinical scenario [20, 60–64].

In summary, we identified common themes across a variety of studies that explored factors influencing use of systematic reviews by policy makers and managers. Useful information has been identified for authors of systematic reviews to inform their preparation of reviews including providing one-page summaries with key messages, tailored to the relevant audience. Moreover, partnerships between researchers and policy makers/managers to facilitate conduct and use of systematic reviews should be considered to enhance relevance of reviews and thereby influence uptake. Finally, these strategies should be rigorously evaluated to determine impact on reviews.

## Additional files

Additional file 1: **Literature search strategy used for Medline; additional search strategies available from the authors.**

Additional file 2: **Barriers and facilitators to use of systematic review, categorised by study.**
