# Supplementary material for: Barriers and facilitators to uptake of systematic reviews by policy makers and health care managers: a scoping review
Source: Implement Sci. 2016 Jan 12;11:4. doi: 10.1186/s13012-016-0370-1 (PMC4709874; doi:10.1186/s13012-016-0370-1)
Supplement: Supplementary file 1 — Literature search strategy used for Medline; additional search strategies available from the authors. [file 13012_2016_370_MOESM1_ESM.docx]

**Appendix 1.** MEDLINE Search Strategy

Formatting of Systematic Reviews

Database: Ovid MEDLINE(R), Ovid MEDLINE(R) In-Process & Other Non-Indexed Citations

Search Strategy:

--------------------------------------------------------------------------------

1 exp Administrative Personnel/

2 exp Decision Making/

3 exp Policy Making/

4 exp Health Policy/

5 ((health or healthcare or hospital*) adj3 (administrator* or analyst* or decisionmak* or decision-mak* or manager* or official$1 or policymak* or policy-mak* or supervisor*)).tw.

6 ((health or healthcare or hospital*) adj (policy or policies)).tw.

7 (policy-mak* or policymak*).tw.

8 (decision-mak* or decisionmak*).tw.

9 ((decision* or policy or policies) adj2 (analys* or analyz* or make or maker* or making or develop*)).tw.

10 or/1-9

11 exp Evidence-Based Practice/

12 exp "Review Literature as Topic"/

13 meta-analysis as topic/

14 ((evidence* or literature or methodologic* or quantitative* or rapid or scoping or systematic*) adj2 (review$1 or overview$1 or synthes*)).ti.

15 (meta-analy* or metanaly* or metaanaly* or meta-regression or metaregression or meta-review* or metareview* or meta-overview* or metaoverview* or meta-synthes* or metasynthes*).ti.

16 (scoping adj (study or studies)).ti.

17 (mapping adj (study or studies)).ti.

18 or/11-17

19 10 and 18

20 ((adapt* or adopt* or appl* or chang* or execut* or implement* or incorporat* or innovat* or interpret* or operational* or practi#e? or practi#ing or transfer* or utilis* or utiliz* or uptake) adj5 (aid or aids or aiding or aided or assist* or bar or barred or barring or barrier* or block* or challeng* or constrain* or deter* or difficult* or discourag* or disincentive* or encourag* or encumber* or encumbranc* or enhanc* or facilitat* or help* or hinder* or hindrance* or impair* or impede* or impeding or impediment* or influen* or interfer* or motivat* or obstruct* or problem* or promot* or restrain* or restrict*)).tw.

21 19 and 20

22 exp Animals/ not (exp Animals/ and Humans/)

23 21 not 22
