# Supplementary material for: Barriers and facilitators to uptake of systematic reviews by policy makers and health care managers: a scoping review
Source: Implement Sci. 2016 Jan 12;11:4. doi: 10.1186/s13012-016-0370-1 (PMC4709874; doi:10.1186/s13012-016-0370-1)
Supplement: Supplementary file 2 — Barriers and facilitators to use of systematic review, categorised by study. [file 13012_2016_370_MOESM2_ESM.docx]

**Additional File 2. Barriers and Facilitators per study**

| **Studies [Reference]** | **Facilitators** | **Barriers** |
| --- | --- | --- |
| Albert 2007 [38] | - Time consuming to search, access and review research findings makes SRs more appealing. | - Policy-makers' belief that searching, accessing and reviewing research findings is highly time consuming is perhaps a good argument for the increased production, promotion and dissemination of systematic reviews - Lack of knowledge and use: No policy-makers mentioned having utilized information from systematic reviews, and most seemed unaware of their existence. |
| Armstrong 2012 [39] | - SRs are useful for: presentation of the review methods and findings at a continuing education session within an organization, presentation of the review at a Board meeting of a national organization whose aim is to promote ergonomics, intended use of the review findings to influence human resource policy within organizations - One respondent indicated that the summary section was the most useful and had been directly used in an internal document for internal information sharing in their department on. - Stakeholders described potential uses of the review as being more indirect (creating a culture); for example for advocacy purposes internally; to promote a particular intervention approach and to identify gaps of where further evaluation was needed. | - One stakeholder commented that the review itself did not appear to be user-friendly due to inaccessible language and dense layout, particularly for practitioners who were not familiar with Cochrane reviews and other systematic reviews |
| Atack 2010 [40] | - Better access to systematic reviews is also needed as this saves time and also increases confidence for decision making, a finding that has been reported elsewhere. | - Although finding the evidence was described as problematic, appraising and synthesizing the evidence was seen as an even bigger challenge. Several Fellows called for greater access to systematic reviews; this was a resource they wanted to see augmented through the desktop. |
| Campbell 2009 [42] | - Most policy makers reported having needed data and reviews in the past 12 months, having commissioned research or reviews during this period, and having used evidence to contribute to the content of policy. - Increasing the opportunities for interaction and exchange between policy makers and researchers is key to promoting the use of research evidence in policy - Involvement in an advisory role by policy makers on research teams (i.e. involved with the development of research questions, assisted with dissemination) - Systematic reviews highly useful (78%) | - Policy makers had difficulty finding brief research summaries and systematic reviews when they were needed (i.e. difficulty accessing SRs) - Policy makers in our sample reported that they often wanted to seek advice from researchers, but sometimes could not find the expertise that they needed, and that they tended to use existing contacts - Researchers perceived that input from policy makers into their research would be of value but were often not sure how best to identify appropriate individuals. |
| Campbell 2011 [41] | - Researchers and policy makers generally found reviews commissioned through Evidence Check to accurately reflect the state of the evidence, implying that the requirement for rigour and comprehensiveness was not unnecessarily compromised by the rapid timeframe in which the reviews were conducted. It is likely that this is due to both knowledge brokers’ attempts to assist in precisely defining the focus and scope of reviews early in the commissioning process, and researchers’ depth of content knowledge and methodological expertise. - Commissioned reviews were mostly perceived by policy makers as useful for decision making. Several policy makers who were involved in formulating a policy response in relation to commissioned reviews believed that the reviews were useful for informing decision making. | NR |
| Ciliska 1999 [21] | - Preferred access to reviews was in full paper format, followed by disk, abstract, summary, and internet - Overviews have the potential to overcome many barriers to research utilization. | - Time - Availability of research results - Resources to implement research - Relevance - Policy climate - provincial/regional - Timeliness - Current practice patterns - Cost of retrieving information - Critical appraisal skills - Credibility of authors of the research - Workplace not supportive of use of research - Insufficient authority to implement research results - Ethical disagreement - Research information not valued at community level |
| Dobbins 2001a (CR to Ciliska 1999) [22] | - Position of end user within organization/system: program manager vs. director vs. medical officer differed in uptake of SRs - Perceived ease of use of SRs: This suggests that the presentation of research evidence is likely as important as the results themselves. - Perception that systematic reviews could overcome the barrier of limited critical appraisal skills: Respondents who perceived systematic reviews could overcome this barrier were 3.4 times more likely to have used a systematic review than those who did not perceive reviews could overcome this barrier - Expecting to use the systematic reviews in the future was highly predictive of use - Respondents who expected to use the reviews in the future were more likely to have used a review than those who did not expect to use the reviews - Respondents who perceived the reviews as being ease to use were three times more likely to use the reviews than those who did not perceived them as ease to use. | NR |
| Dobbins 2001b  (CR to Ciliska 1999) [23] | - The percentage of retrieved articles read in a month - Number of years since graduation - Value the organization placed on using research evidence for decision making - Ongoing training in critical appraisal of research literature - Expecting to use the systematic reviews in the future - Perception that systematic reviews would overcome the barrier of not having enough time to use research evidence - Having direct access to on-line database searching - One’s age - Making decisions in collaboration with other community organizations - Existence of mechanisms to facilitate transfer of new info in health unit | NR |
| Dobbins 2004 [30] | - Opportunities for training and education on systematic reviews (definition, significance, appraisal) | NR |
| Dobbins 2004 [31] | - Executive summary was the most important component of the systematic review, followed by the conclusion (42 percent), abstract (39 percent), and text (36 percent) - Easy to use - Relevance to policy decisions - recognition of relative importance of SR compared to other sources of information (culture of evidence based decision making) - Concrete recommendations for practice. | NR |
| Dobbins 2007 [43] | - Saves time and gives them more confidence knowing their decisions are based on the culmination of many studies instead of just a few - Electronic communication preferred - Executive summaries of research - Newsletters with summaries of research directly emailed to them - One-to-one interaction with the researcher to discuss research findings - Provide guidance and suggestions for implementation of findings, not just reporting facts | - Information overload - limited time to read full study reports |
| Dobbins 2009a [24] | - organizational research culture - Tailored, targeted messages - Website informational material - Active delivery of information (as opposed to access to online registry) | - Information overload - Limited time to find, retrieve, read and translate research |
| Jewell 2008 [44] | - Delineating the effects for a particular group with more focused subgroup analyses in SRs - For example, particular kinds of research evidence are especially resonant in the political context, namely, evidence that is able to concretize impact. General health arguments tended to be less effective than those asserting particular benefits or harms. Concise statements about lives or money can infuse the political discussion with a tone of rationality, framing the trade-offs as technical and straightforward. - It must be packaged to incite and persuade, “to translate the evidence into something that is understandable by the average legislator, average citizen.” delineating the effects for a particular group with more focused subgroup analyses in SRs - One official thought the best change that could be made to facilitate evidence utilization would be a bullet-point evaluation or rating system of study design quality so that “for those of us who don’t make our living doing that, we don’t have to read a half dozen pages to ferret it out.” | - Administrators: limited understanding of the function of SRs - SRs not addressing relevant policy issues (not targeted) - Lack of expertise in evaluating SRs - Attacks on an Evidence-Based Approach. Several officials also discussed instances in which the whole notion of evidence-based health care had come under direct attack, usually by pharmaceutical companies, sometimes in collaboration with advocacy groups, some of which hid their involvement with industry. - The health agency staff had not been taught “to continue to use research to inform their decisions, to inform their practice.” They therefore made decisions based on “common sense,” “gut level,” “standards of practice,” and comparative convenience and awareness of available data, rather than based on systematic reviews of research - Limited quantity of research on topics of importance to them, e.g. economic impact, emerging technologies - Lack features that would make them easier for government officials to evaluate. - Systematic reviews do not necessarily frame the existing evidence in terms of their policy applications - Existing studies and systematic reviews commonly lack features that would make them easier for government officials to evaluate. For example, the quality of studies is often difficult for nonexperts to interpret because the explanation of research methods is long and complicated. - Accessibility. Even when evidence is available, policymakers may have problems obtaining it. |
| Lavis 2005 [45] | - Removing jargon and using language that is locally applicable, engage in discussion about the potential implications of the review - Fund production and updating of SRs with additional resources for health care managers and policy makers to interact, fund local adaptation process for SR - Ensure SRs are included in a one-stop-shop that provides quality-appraised reviews - Reallocate funding away from single study K transfer strategies, fund rapid reviews, more proactive k transfer, health care managers - Identify completed systematic reviews that address questions that are now or soon to be relevant - Develop a more user-friendly ‘front end’ for potentially relevant systematic reviews (e.g. one page of take-home messages and a three-page executive summary) to facilitate rapid assessments of the relevance of a review by health care managers and policy-makers and, when the review is deemed highly relevant, more graded entry into the full details of the review - Providing information about the benefits, harms (or risks), and costs was supported by the five health care managers and the 15 health care policy-makers - Identify attributes of the context in which the research included in a systematic review was conducted to inform assessments of the applicability of the review in other contexts - Add additional local value to any type of systematic review by using language that is locally applicable and by engaging in discussions about the implications of reviews with the health care managers and policy-makers who could potentially act on the reviews’ take-home messages - Make the user-friendly ‘front end’ of systematic reviews available through an online database that could be searched using keywords that make sense to health care managers and policy-makers and that is linked to the full reviews when they are available through other sources, such as The Cochrane Library. | - Avoid providing specific recommendations for action based on systematic review |
| Packer 2000 [52] | - Meeting requestors time constraints - Ease of access - reassurance that no reviews have been missed - Considering a local interpretation of the evidence - Consistency in follow-up - ensure user organizations understand the nature of the service (on demand access, appraisal, and expert opinion on existing reviews of evidence) - Providing training in basic search skills. | - Policy decisions are made based on other factors like cost and equity considerations, particularly if evidence base is frail - If department within commissioning organization is not in a position of strength, unlikely that evidence will be used for decision-making - Lacking skills to critically appraise papers found in a lit search - Lack of time to find or discuss evidence, usually need an answer to a problem on the same day - Health authority is not interested in the methods used by the public health directorate to access and appraise the evidence - Reviews covered issues at a more complex level than required |
| Ritter 2009 [46] | NR | - Academic literature is nuanced and complex and frequently contradictory |
| Rosenbaum 2011 [47] | - perception that reviews facilitate critical appraisal of evidence and are easy to use, information about what wors and clearly articulated implications for policy (costs, applicability, impacts on equity) - Well written and concise - Coming from credible sources - References are clear - Section on the relevance of the evidence and the intervention for LMICs - Table describing the characteristics of the reviews: makes clear what the review was looking for - Liked the framing of the title as a question - Simplifying the text and tables, and ensuring that the results in the text matched those in the tables - Limiting the number of tables and not letting them break across pages - Use of familiar, non-jargon language recommended - Eliminating abbreviations - Using consistent language and standard phrases to describe effect sizes and the quality of the evidence - Replacing unfamiliar terms or adding definitions - Moving partner logos and the summary publication date to the front page - Timely retrieval of relevant research | - Mismatch between the type of content offered and their information needs - Policymakers expected content lying outside the scope of a review: recommendations, outcome measurements not usually included in a review, detailed information about local applicability or costs and a broader framing of the research enquiry - not comprehensive enough - Wanted a shorter, clearer presentation - Not have time to read a long document - Difficult to understand by people not trained in evidence-based medicine - Concepts presented in tables, including those that showed the GRADE assessment and different levels of risk, were not clear - Words like ‘sample size’ and ‘relative risk’ would be difficult to interpret - Tables running over two pages were cumbersome to read - Abbreviations caused confusion - -Numbers in the text and those in the tables do not correspond precisely - Use of jargon and/or unfamiliar vocabulary - Reduced interest in the content when they discovered that the quality of the evidence was low, that no evidence for important outcomes existed or that the studies were old - Participants’ poor conceptual understanding of systematic reviews - Research often published in academic sources, poorly accessible to policy makers, LMIC policy makers have limited access to subscription-based K or the internet |
| Shepperd 2013 [48] | NR | - Policy inconsistency within health care system (differing interests, different policies run in parallel, performance based incentives) - Problems with the production of knowledge and information overload. The challenge of multiple sources of information compiled by national bodies is significant and can mean that the practical utility of evidence is lost. Clinicians and commissioners reported being bombarded with lists, guidance and evidence. - Translating evidence to the local context (including sub groups of patients): Systems/structures to link evidence to current practice as lacking/evolving: Individuals frequently had to make independent decisions about how to relate evidence to the needs of their local context, discuss and debate the evidence with local stakeholders and take decisions about its use in practice. - Patient or parent expectations. - The lack of an explicit framework can result in strong resistance and skepticism from clinicians, can lead to ambiguity within the health system and can create tension between clinicians and commissioners. |
| Suter 2011 [49] | - One page summaries in plain language (with reference to more detailed findings so the reader is able to investigate further if needed) - A list of questions to be considered when developing and implementing an integrated health system, information about how to engage stakeholders, build relationships and communicate appropriately across target audiences - more information about integration for rural and remote communities - Use of stories to help integration come alive for participants so they could see how it lives operationally - Use of less dense and more accessible articles - collaborative creation of knowledge in a format that is easy to view and comprehensible, and allows fast and easy referencing - Use of knowledge synthesis (in this case 10 universal principles of integrated health care systems) to assist and inform integration efforts | - Tended not to use the full report instead referring to the less dense, more accessible articles - Preference for less dense, more accessible literature, and one page summaries with references, so the reader is able to investigate further, and case studies - Lack of detail on how to use strategies, tools, processes that would lead to successful integration (i.e. guidance on breaking down systems barriers, or how to achieve integration in the context of big, complex system) |
| Vogel 2013 [50] | NR | - Lack of relevant systematic reviews |
| Yousefi-Nooraie 2009 [51] | - Access to international research - Priority of and support for systematic reviews - Competency and willingness of researchers to conduct systematic reviews - Willingness and competency of health care providers to use systematic reviews - Quality of local primary research - Visibility and access to local research - training of professional methodologists in the conduct of systematic review related fields - Quality of conduct, reporting of local primary research - Teaching about systematic reviews, presenting selected important systematic reviews to policy makers may change their attitudes towards evidence-based decision making, presenting successful/unsuccessful policies, integration of SR course into postgraduate educational curriculae, mandatory education of research methods to researchers, consultation support in methodology and scientific writing, professional methodologists on research teams - Involvement of librarians and health information specialists as a solution to lack of database access, establishment of a national portal for expanding access - Give higher funding scores to SR in assessment and promotion of faculty, higher priority by research councils, announce priorities to be addressed using SR, new journals specializing on the topic, editors should take account whether SR has been used to indicate the need for the study | - Changing policy makers' attitudes - use of data collection tools that are unvalidated; quality control mechanisms for research tools lacking, as is the establishment of national bank of valid tools in local language - Lack of indexing local journals in international databases, harmonized reporting criteria, editorial processes and presentation of local journals, minimum standards for reporting of research for all local journals. Coverage and searching quality of databases of papers published in local language needs improvement, single national database for research registration, technical and professional support for current databases |
|  |  |  |

**Abbreviations**: NR= not reported; SR, systematic review; CR, companion report.
